# Butyrate protects and synergizes with nicotine against iron- and manganese-induced toxicities in cell culture: Implications for neurodegenerative diseases

**DOI:** 10.21203/rs.3.rs-3389904/v1

**Published:** 2023-10-05

**Authors:** Yousef Tizabi, Bruk Getachew, Michael Aschner

**Affiliations:** Howard University College of Medicine; Howard University College of Medicine; Albert Einstein College of Medicine

**Keywords:** Metal toxicity, Parkinson’s disease, SH-SY5Y cells, Short Chain Fatty Acids, Nicotinic Receptors, Free Fatty Acid Receptors

## Abstract

Toxic exposures to heavy metals, such as iron (Fe) and manganese (Mn), can result in long-range neurological diseases and are therefore of significant environmental and medical concerns. We have previously reported that damage to neuroblastoma-derived dopaminergic cells (SH-SY5Y) by both Fe and Mn could be prevented by pre-treatment with nicotine. Moreover, butyrate, a short chain fatty acid (SCFA) provided protection against salsolinol, a selective dopaminergic toxin, in the same cell line. Here, we broadened the investigation to determine whether butyrate might also protect against Fe and/or Mn, and whether, if combined with nicotine, an additive or synergistic effect might be observed. Both butyrate and nicotine concentration-dependently blocked Fe and Mn toxicities. The ineffective concentrations of nicotine and butyrate, when combined, provided full protection against both Fe and Mn. Moreover, the effects of nicotine but not butyrate could be blocked by mecamylamine, a non-selective nicotinic antagonist. On the other hand, the effects of butyrate, but not nicotine, could be blocked by beta-hydroxy butyrate, a fatty acid-3 receptor antagonist. These results not only provide further support for neuroprotective effects of both nicotine and butyrate but indicate distinct mechanisms of action for each one. Furthermore, potential utility of the combination of butyrate and nicotine against heavy metal toxicities is suggested.

## Introduction

Heavy metals in general, and iron (Fe) and manganese (Mn) in particular, play crucial roles in various biological functions. Specifically, Fe is a critical component of a variety of enzymes or co-enzymes, including catalases and cytochromes that mediate cellular processes and drug metabolism. Fe is well recognized as an essential element in hemoglobin synthesis, where its deficiency is reflected in widespread Fe-deficiency anemia (Anand and Gupta 2018). Similarly, Mn, an activator or cofactor for a variety of metalloenzymes, is a transition metal that is essential for normal cell growth and development (Yousefi Babadi et al. 2014; Caito and Aschner 2015; O’Neal and Zheng 2015; Peres et al. 2016; Andrade et al. 2017). Moreover, the enzyme or co-enzymes utilizing Mn play key roles in such functions as gluconeogenesis, suppression of oxidative stress (Mn-superoxide dismutase, SOD) and conversion of glutamate into glutamine (glutamine synthetase) (Aschner and Gannon 1994; Burton and Guilarte 2009), all of which have critical biological functions. Thus, Mn deficiency may lead to varied maladies, including impaired reproductive function, retarded growth, skeletal abnormalities, and seizures (Roth and Garrick 2003; Aschner and Aschner 2005).

On the other hand, excessive exposure to Fe or Mn, may also have serious detrimental consequences such as reproductive impairment, diabetes, increased infection risk as well as causing neurotoxicity leading to Parkinson’s disease-like syndrome (McMillan 1999; Peres et al. 2016; Andrade et al. 2017; Wang et al. 2016; Zhang and Gan 2017; Ganz 2018). Regarding the latter, both Fe and Mn can selectively damage the dopaminergic neurons in the substantia nigra, leading to parkinsonism (Dexter et al. 1989; Riederer et al. 1989; Lu et al. 2023; Mateo et al. 2023). Parkinsonism may be caused one or more small strokes; in which case it is referred to as vascular parkinsonism, or by toxins. It is characterized by motor syndrome that manifests as rigidity, tremors, and bradykinesia, like what is observed in Parkinson’s disease (PD), which is caused by a gradual loss of nerve cells in substantia nigra pars compacta (SN_pc_). One major distinction between the two is that unlike PD, parkinsonism is not progressive (Shrimanker et al. 2023). Nonetheless, the search for slowing or stopping the progression of PD is an ongoing challenge as its etiology remains elusive. The most common treatment of PD consists of dopamine replacement (e.g., levodopa = L-DOPA), which not only loses its full efficacy in a few years but may also induce severe dyskinesia (Picconi et al. 2017; Tizabi et al. 2021b). Hence additional studies on etiology as well as development of more efficacious interventions aiming at neuroprotection are urgently needed (Tizabi et al. 2021a). Since excess accumulation of trace elements such as Fe and Mn have been suggested to play a role in PD etiology (Tanner et al. 2011; Wang et al. 2016; Andrade et al. 2017; Vaccari et al. 2017), drugs that might inhibit or block the toxicities of these heavy metals, may not only be of use in detoxification of these elements, but might point to novel therapeutics for PD.

We have reported recently that toxicity to the neuroblastoma-derived dopaminergic SH-SY5Y cells induced by both Fe and Mn could be prevented by pre-treatment with nicotine (Getachew et al. 2019). Moreover, both nicotine and butyrate, a short chain fatty acid (SCFA) provided protection against salsolinol (SALS), a selective dopaminergic toxin, in the same cell line (Copeland et al. 2005; Getachew et al. 2020). Butyrate, acting as an energy source for colonic epithelial cells, has anti-inflammatory, enteroendocrine and epigenetic effects that not only can influence colonic and systemic health, but can also affect the brain functions (Cantu-Jungles et al. 2019). Indeed, a few studies indicate beneficial effects of butyrate in animal models of PD (Laurent et al. 2013; Liu et al. 2017). However, the exact mechanism of action of butyrate in the central nervous system remains far from complete. Here, we investigated whether butyrate might also offer protection against Fe and/or Mn toxicities, and whether it might have an additive or synergistic effect when combined with nicotine.

## Material and Methods

Butyrate, beta-hydroxy butyrate (BHB), a selective FA3R antagonist (Kimura et al. 2011; Ulven 2012; Inoue et al. 2014), SALS, manganese sulfate, iron sulfate, nicotine (bitartrate salt), mecamylamine (MEC), a non-selective nicotinic antagonist, and other analytical reagents were purchased from Sigma Chemical Company (Sigma-Aldrich, St. Louis, MO). The SH-SY5Y human-derived neuroblastoma cell line was purchased from American Type Culture Collection (ATCC, Manassas, VA). 3, (4,5-dimethylthiazol-2-yl)-2,5- diphenyltetrazolium bromide (MTT) reagent was purchased from Fisher Scientific (Pittsburgh, PA).

As reported in detail previously (Getachew et al. 2019, 2020) and briefly here, SH-SY5Y cells were cultured in a 1:1 mixture of Dulbecco Modified Eagle Medium (DMEM) and Ham’s F12 supplemented with 10% fetal bovine serum, penicillin/streptomycin (100 IU/ml), and gentamicin (50ug/ml) at 37^0^ C in 95% O_2_/5% CO_2_ humidified incubator. The cells were trypsinized when confluent and plated in 96 well plates (4.2 x 10^4^ cells/well). Cells were allowed to adhere to bottom surface for 24 h. Then, fresh media containing various concentrations of Fe, Mn, butyrate, nicotine, DHB, and nicotinic antagonists were added to the carefully aspirated wells.

We used the toxic concentrations (60 μM) of both Fe and Mn, where approximately 40–42% cell death was induced following 24 h incubation with either metal. These concentrations were based on more recent reports (Francisco et al. 2021; Zhang N. et al. 2022) and our previous studies (Getachew et al. 2019) where we had observed about 30 percent toxicity with lower concentrations of 20–40 μM of each metal. Hence, we chose a higher concentration of both Fe and Mn to induce significantly more toxicity to allow even subtle protections by our treatments. We then determined concentration-responses for butyrate and nicotine against both Fe and Mn toxicities. The reason for including various concentrations of nicotine was to be able to determine potential additive or synergistic effects of combination treatments. Concentrations of MEC and DHB were based on our previous results (Getachew et al. 2018, 2019).

Butyrate and nicotine, alone or in combination, were added 1 h prior to Fe or Mn. We chose a wide range butyrate concentration to determine optimal protective concentration. For nicotine, we followed our previous concentration range (Getachew et al. 2019). The antagonists, MEC and DHB were added 1 h prior to nicotine or butyrate. In all cases, the control group consisted of cells that were maintained in media alone and without any drug treatment. All treatments were carried out for 24 h and the effect on cell viability was determined following the 24 h incubation. Each cell viability study was run in sextuplicate (i.e., 6 replicates) and a minimum of 4 assays were conducted for each experimental manipulation.

Determination of cell viability was done by 3, (4,5-dimethylthiazol-2-yl)-2,5-diphenyltetrazolium bromide (MTT) colorimetric assay according to the manufacturer’s protocol as described previously (Getachew et al. 2019, 2020). Briefly, the yellow MTT tetrazolium salt (0.5 mg/ml) was dissolved in phosphate-buffered saline (PBS) with 10 mM (4-(2-hydroxyethyl)-1-piperazineethanesulfonic acid (HEPES). 30 μl of MTT was added to each well and incubated for 3 h at 37° C. The live cells cause a reduction of the yellow salt to insoluble purple formazan crystals. The wells were then aspirated, and 50 μl of dimethyl sulfoxide (DMSO) was added to the wells to solubilize the crystals, and the plates were placed in a shaker for an hour. The plates were then read spectrophotometrically at 570 nm with a background of 630 nm in a plate reader. Cell viability was determined by subtracting the test results from the background and is presented as a percentage of the control.

Data are expressed as mean ± standard error of the mean (SEM). Statistical differences within and between treatment groups were determined by one-way analysis of variance (ANOVA) followed by post- hoc Newman–Keuls Multiple comparison test, where P< 0.05 was considered statistically significant. Data were analyzed using Graphpad Prism 7 (Graphpad Software, Inc., San Diego, CA).

## Results

**Figure 1:**
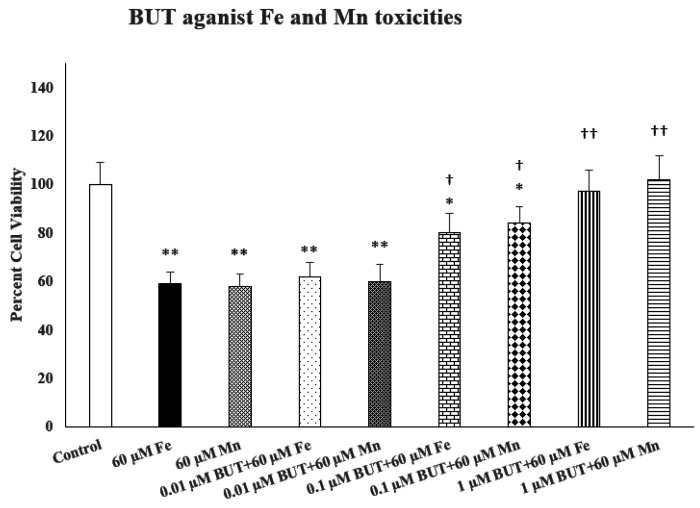
Effects of various concentrations of butyrate (BUT) against iron (Fe)- and manganese (Mn)-induced toxicities. Cells were treated with Fe, Mn with and without BUT for 24 h and cell viability was determined by MTT. BUT was added 1 h before Fe or Mn. Values are mean ± SEM. **p<0.01 compared to control. ^†^p<0.05, ^††^p<0.01 compared to Fe or Mn only. N = 5 per treatment.

Figure 1 depicts the effects of various concentrations of butyrate (0.01–1.0 –M) against both Fe- and Mn-induced toxicities in SH-SY5Y cells. For both metals, we used a concentration of 60 μM because at this concentration significant toxicity (about 40%) is observed during the 24 h exposure. As seen, there was a concentration-dependent protection by butyrate against both iron [F(4,20) = 11.4, p < 0.01] and manganese [F(4,20) = 12.3, p < 0.0] toxicities with full protection at 1.0 μM butyrate. Butyrate by itself, at any concentration, did not affect the cell viability (data not shown)

Effects of various concentrations of butyrate (BUT) against iron (Fe)- and manganese (Mn)-induced toxicities. Cells were treated with Fe, Mn with and without BUT for 24 h and cell viability was determined by MTT. BUT was added 1 h before Fe or Mn. Values are mean ± SEM. **p < 0.01 compared to control. ^†^p < 0.05, ^††^p < 0.01 compared to Fe or Mn only. N = 5 per treatment.

**Figure 2:**
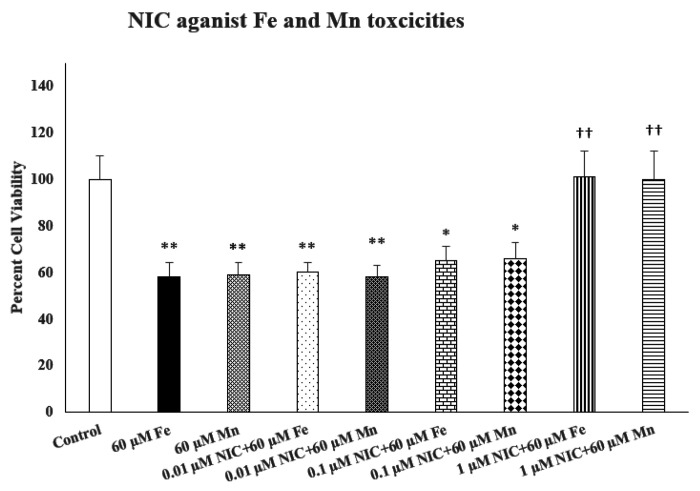
Effects of various concentrations of nicotine (NIC) against iron (Fe)- and manganese (Mn)-induced toxicities. Cells were treated with Fe, Mn with and without NIC for 24 h and cell viability was determined by MTT. NIC was added 1 h before Fe or Mn. Values are mean ± SEM. **p<0.01 compared to control. ^†^p<0.05, ^††^p<0.01 compared to Fe or Mn only. N = 5 per treatment.

Figure 2 depicts the effect of various concentrations of nicotine (NIC) (0.01–1.0 μM) against Fe- and Mn- induced toxicities. Here also, there was a concentration-dependent protection by Nic [F(4,20) = 10.4, p < 0.01], similar to what we had observed previously (Getachew et al. 2019). This experiment was carried out to determine the concentrations of nicotine in these batch of cells to allow combination studies with butyrate

Effects of various concentrations of nicotine (NIC) against iron (Fe)- and manganese (Mn)-induced toxicities. Cells were treated with Fe, Mn with and without NIC for 24 h and cell viability was determined by MTT. NIC was added 1 h before Fe or Mn. Values are mean ± SEM. **p < 0.01 compared to control. †p < 0.05, ††p < 0.01 compared to Fe or Mn only. N = 5 per treatment.

**Figure 3:**
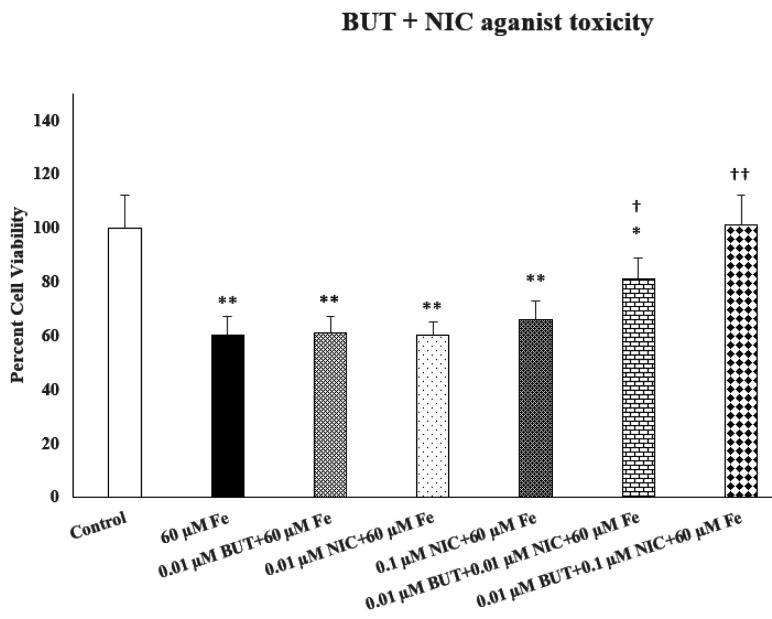
Effects of combination of nicotine (NIC) and butyrate (BUT) against iron (Fe)-induced toxicity. Cells were treated with 2 ineffective concentrations of NIC and one ineffective concentration of BUT for 24 h. For each combination, NIC and BUT were added together 1 h before Fe. Cell viability was determined by MTT. Values are mean ± SEM. *p<0.05, **p<0.01 compared to control. ^†^p<0.05, ^††^p<0.01 compared to Fe only. N = 5 per treatment.

Figure 3 depicts the effect of combination of an ineffective concentration of butyrate (BUT) with 2 ineffective concentrations of nicotine (NIC) against iron (Fe)-induced toxicity. As seen, the combination of the lowest NIC concentration with the ineffective concentration of BUT resulted in substantial protection (approximately 75%. P < 0.05) against Fe toxicity. The combination of higher ineffective concentration of NIC with the same concentration of BUT resulted in 100% protection (p < 0.01). Hence, a synergistic protection by the combination of ineffective concentrations of NIC and BUT against Fe-induced toxicity can be suggested.

Effects of combination of nicotine (NIC) and butyrate (BUT) against iron (Fe)-induced toxicity. Cells were treated with 2 ineffective concentrations of NIC and one ineffective concentration of BUT for 24 h. For each combination, NIC and BUT were added together 1 h before Fe. Cell viability was determined by MTT. Values are mean ± SEM. *p < 0.05, **p < 0.01 compared to control. †p < 0.05, ††p < 0.01 compared to Fe only. N = 5 per treatment.

**Figure 4:**
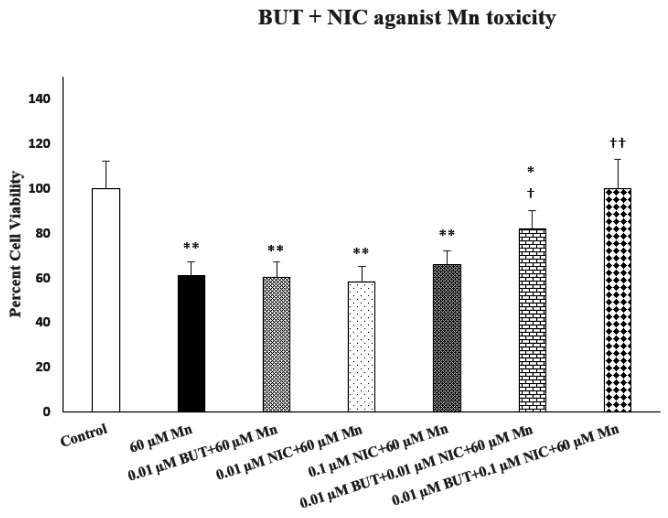
Effects of combination of nicotine (Nic) and butyrate (BUT) against manganese (Mn)-induced toxicity. Cells were treated with 2 ineffective concentrations of Nic and one ineffective concentration of BUT for 24 h. For each combination, Nic and BUT were added together 1 h before Mn. Cell viability was determined by MTT. Values are mean ± SEM. *p<0.05, **p<0.01 compared to control. ^†^p<0.05, ^††^p<0.01 compared to Mn only. N = 5 per treatment.

Figure 4 depicts the effect of combination of ineffective concentration of butyrate (BUT) with 2 ineffective concentrations of nicotine (NIC) against manganese (Mn)-induced toxicity. As seen, the combination of the lowest NIC concentration with the ineffective concentration of BUT resulted in substantial protection (approximately 76%. P < 0.05) against Mn toxicity. The combination of higher ineffective concentration of NIC with the same concentration of BUT resulted in 100% protection (p < 0.01). Hence, a synergistic protection by the combination of ineffective concentrations of NIC and BUT against Mn-induced toxicity can be suggested.

Effects of combination of nicotine (Nic) and butyrate (BUT) against manganese (Mn)-induced toxicity. Cells were treated with 2 ineffective concentrations of Nic and one ineffective concentration of BUT for 24 h. For each combination, Nic and BUT were added together 1 h before Mn. Cell viability was determined by MTT. Values are mean ± SEM. *p < 0.05, **p < 0.01 compared to control. †p < 0.05, ††p < 0.01 compared to Mn only. N = 5 per treatment.

**Figure 5:**
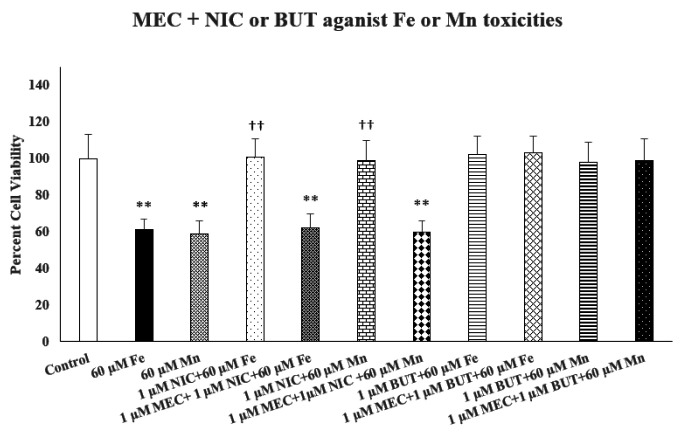
Effect of mecamylamine (MEC) on nicotine (NIC) or butyrate (BUT) protection against iron (Fe) or manganese (Mn) toxicities. MEC was added 1 h before NIC or BUT, which in turn, were added 1 h prior to Fe or Mn. Cell viability was assessed using MTT assay 24 h later. Values are mean ± SEM. **p<0.01 compared to control, ^††^p<0.01 compared to Fe or Mn only. N = 5 per treatment

Figure 5 depicts the effect of mecamylamine (MEC), a non-selective nicotinic receptor antagonist on protective effects of nicotine (NIC) or butyrate (BUT) against iron (Fe)- or manganese (Mn)-induced toxicities. As seen, MEC (1.0 μM) completely blocked the protective effects of NIC against both Fe- and Mn- induced toxicities (p< 0.01). On the other hand, MEC did not affect the protective effect of BUT against either Fe- or Mn-induced toxicities, suggesting selective action of NIC via nicotinic receptor.

Effect of mecamylamine (MEC) on nicotine (NIC) or butyrate (BUT) protection against iron (Fe) or manganese (Mn) toxicities. MEC was added 1 h before NIC or BUT, which in turn, were added 1 h prior to Fe or Mn. Cell viability was assessed using MTT assay 24 h later. Values are mean ± SEM. **p < 0.01 compared to control, ††p < 0.01 compared to Fe or Mn only. N = 5 per treatment

**Figure 6:**
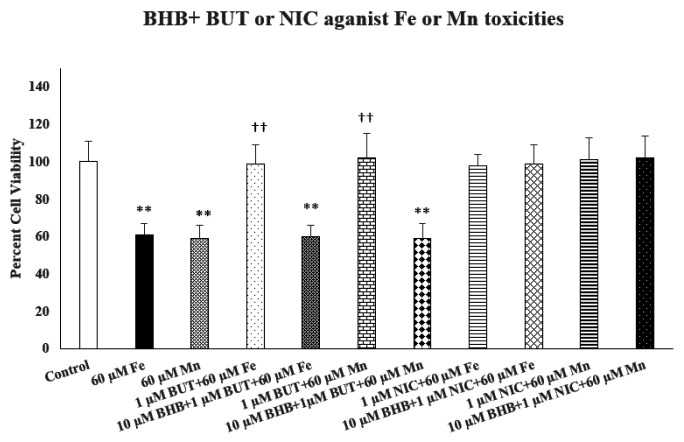
Effect of beta-hydroxy butyrate (BHB), a selective fatty acid 3 receptor (FA3R) antagonist on nicotine (NIC) or butyrate (BUT) protection against iron (Fe)- or manganese (Mn)-induced toxicities. BHB was added 1 h before Nic or BUT, which in turn, were added 1 h prior to Fe or Mn. Cell viability was assessed using MTT assay 24 h later. Values are mean ± SEM. **p<0.01 compared to control, ^††^p<0.01 compared to Fe or Mn only. N=5 per treatment.

Figure 6 depicts the effect of beta-hydroxy butyrate (BHB), a selective fatty acid 3 receptor (FA3R) antagonist, on protective effects of nicotine (NIC) or butyrate (BUT) against iron (Fe)- or manganese (Mn)- induced toxicities. As seen, BHB (10 μM) completely blocked the protective effects of BUT against both Fe and Mn (p < 0.01). On the other hand, BHB did not affect the protective effect of Nic against either Fe or Mn, suggesting selective action of BUT through FA3R.

BHB (10 μM) by itself did not have any effect on cell viability (data not shown).

Effect of beta-hydroxy butyrate (BHB), a selective fatty acid 3 receptor (FA3R) antagonist on nicotine (NIC) or butyrate (BUT) protection against iron (Fe)- or manganese (Mn)-induced toxicities. BHB was added 1 h before Nic or BUT, which in turn, were added 1 h prior to Fe or Mn. Cell viability was assessed using MTT assay 24 h later. Values are mean ± SEM. **p < 0.01 compared to control, ††p < 0.01 compared to Fe or Mn only. N = 5 per treatment.

## Discussion

The result of this study provide evidence for neuroprotective effects of butyrate against both Fe and Mn toxicities in neuroblastoma-derived dopaminergic cells. Moreover, the combination of butyrate with nicotine may provide synergistic protection against toxicities of these 2 heavy metals.

It is well-established that excessive exposure to either Fe or Mn can result in Parkinson or Parkinson-like symptoms referred to as parkinsonism. Thus, efforts to prevent toxicity induced by excess of these essential metals can also prevent the neurodegenerative consequences. We had reported previously that nicotine may be considered a strong and widely applicable neuroprotective agent as it shows protection not only against Fe- and Mn-induced toxicities but also a variety of other endogenous or exogenous agents such as SALS, aminochrome, and rotenone that selectively damage the dopaminergic neurons (Muñoz et al. 2012; Getachew et al. 2019; Tizabi et al. 2021a). Butyrate protection against SALS in *in-vitro* models have also been demonstrated (Getachew et al. 2020). Furthermore, based on beneficial effects of butyrate, including its attenuation of motor impairments in various rat models of PD (e.g., those induced by 6-hydroxydopamine, rotenone or MPTP), its applicability as a neuroprotectant in PD has been suggested (Laurent et al. 2013; Liu et al. 2017; Srivastav et al. 2019).

Whereas nicotine acts via nicotinic cholinergic receptors, butyrate action is mediated primarily via FA3Rs. Further verification of the distinct mechanisms of action of nicotine and butyrate in our model was provided by the results showing complete block of nicotine effect by mecamylamine, a blocker of nicotinic receptors, and complete block of butyrate by BHB, a blocker of FA3R. Additionally, it was shown neither mecamylamine affects butyrate’s protection, nor BHB affects nicotine’s protection against either Fe or Mn toxicities. Butyrate may also act as histone deacetylase (HDAC) inhibitor but stimulator of Nrf2/HO-1 axis and glucagon like peptide-1 (Liu et al. 2017; Funakohi-Tago et al. 2018; Cantu-Jungles et al. 2019). Nonetheless, both nicotine and butyrate possess anti-inflammatory properties, conferring on them therapeutic potentials in neuroinflammatory-associated diseases including neurodegenerative and/or neuropsychiatric disorders (Hurley and Tizabi 2013; Tizabi 2016; Tizabi et al. 2021a).

Since both nicotine and butyrate show anti-inflammatory and neuroprotective properties, albeit via different mechanisms, it may be suggested that their combination be considered as a superior intervention than each alone. For example, effectiveness of nicotine (AlQasrawi et al. 2020; Zhang W. et al. 2022) and butyrate (Chen et al. 2020; Silveira et al. 2023; Recharla et al. 2023) alone in inflammatory bowel disease, have been suggested, hence, their combination might provide more effective intervention. Furthermore, as butyrate has a low bioavailability (Witt et al. 2003; Ghosh et al. 2012), combining it with nicotine would permit use of a higher concentration of it and hence a better therapeutic outcome. This contention is supported by our current results, where ineffective concentrations of nicotine and butyrate when combined, provided protection against both Fe- and Mn-induced toxicities.

Finally, interaction of Fe, Mn, butyrate, and nicotine with gut microbiota have been recently highlighted (Tinkov et al. 2021; Whitehead et al. 2022; Nicese et al. 2023; Xiao et al. 2023). Hence, potential exploits of the gut-brain axis in amelioration of heavy metal toxicities and/or prevention or delay of neurodegenerative diseases is worth consideration. In this regard, butyrate, with demonstrated probiotic effect as well novel compounds that could synergize with nicotine and/or butyrate in promoting beneficial gut bacteria and suppressing the opportunistic ones could open new therapeutics not only in metal toxicity but also neurodegenerative diseases.

In summary, our results not only support neuroprotective effects of nicotine and butyrate in countering Fe and Mn toxicities but indicate a synergistic protection by combination of the two. Moreover, distinct mechanisms of action for each metal, i.e., nicotinic receptor for nicotine and FA3R for butyrate are indicated. Further exploitation of mechanisms of action of butyrate and nicotine may provide novel targets for metal toxicities and/or amelioration of neurodegenerative diseases.

## Data Availability

Not applicable

## Highlights

- Iron (Fe) and manganese (Mn) cause significant toxicity in SH-SY5Y cells.
- Nicotine and butyrate each can completely block the toxicities of both Fe and Mn.
- Nicotine effect is mediated via nicotinic receptors.
- Butyrate effect is mediated via fatty acid-3 receptor.
- Butyrate and nicotine provide synergistic protection against toxicities of Fe and Mn.

## Abbreviations

BHB: beta-hydroxy butyrate
BUT: butyrate
FA3R: fatty acid 3 receptor
Fe: iron
HDAC: histone deacetylase
L-DOPA: levodopa
MEC: mecamylamine
Mn: manganese
NIC: nicotine
PD: Parkinson’s disease
SALS: salsolinol
SCFA: short chain fatty acid
SEM: standard error of the mean
SN_pc_: substantia nigra pars compacta
SOD: superoxide dismutase
